# Automatic International Classification of Diseases Coding System: Deep Contextualized Language Model With Rule-Based Approaches

**DOI:** 10.2196/37557

**Published:** 2022-06-29

**Authors:** Pei-Fu Chen, Kuan-Chih Chen, Wei-Chih Liao, Feipei Lai, Tai-Liang He, Sheng-Che Lin, Wei-Jen Chen, Chi-Yu Yang, Yu-Cheng Lin, I-Chang Tsai, Chi-Hao Chiu, Shu-Chih Chang, Fang-Ming Hung

**Affiliations:** 1 Graduate Institute of Biomedical Electronics and Bioinformatics National Taiwan University Taipei Taiwan; 2 Department of Anesthesiology Far Eastern Memorial Hospital New Taipei City Taiwan; 3 Department of Internal Medicine Far Eastern Memorial Hospital New Taipei City Taiwan; 4 Department of Computer Science and Information Engineering National Taiwan University Taipei Taiwan; 5 Department of Electrical Engineering National Taiwan University Taipei Taiwan; 6 Department of Information Technology Far Eastern Memorial Hospital New Taipei City Taiwan; 7 Section of Cardiovascular Medicine, Cardiovascular Center Far Eastern Memorial Hospital New Taipei City Taiwan; 8 Department of Pediatrics Far Eastern Memorial Hospital New Taipei City Taiwan; 9 Department of Healthcare Administration Oriental Institute of Technology New Taipei City Taiwan; 10 Artificial Intelligence Center Far Eastern Memorial Hospital New Taipei City Taiwan; 11 Section of Health Insurance, Department of Medical Affairs Far Eastern Memorial Hospital New Taipei City Taiwan; 12 Medical Records Department Far Eastern Memorial Hospital New Taipei City Taiwan; 13 Department of Medical Affairs Far Eastern Memorial Hospital New Taipei City Taiwan; 14 Department of Surgical Intensive Care Unit Far Eastern Memorial Hospital New Taipei City Taiwan

**Keywords:** deep learning, International Classification of Diseases, medical records, multilabel text classification, natural language processing, coding system, algorithm, electronic health record, data mining

## Abstract

**Background:**

The tenth revision of the International Classification of Diseases (ICD-10) is widely used for epidemiological research and health management. The clinical modification (CM) and procedure coding system (PCS) of ICD-10 were developed to describe more clinical details with increasing diagnosis and procedure codes and applied in disease-related groups for reimbursement. The expansion of codes made the coding time-consuming and less accurate. The state-of-the-art model using deep contextual word embeddings was used for automatic multilabel text classification of ICD-10. In addition to input discharge diagnoses (DD), the performance can be improved by appropriate preprocessing methods for the text from other document types, such as medical history, comorbidity and complication, surgical method, and special examination.

**Objective:**

This study aims to establish a contextual language model with rule-based preprocessing methods to develop the model for ICD-10 multilabel classification.

**Methods:**

We retrieved electronic health records from a medical center. We first compared different word embedding methods. Second, we compared the preprocessing methods using the best-performing embeddings. We compared biomedical bidirectional encoder representations from transformers (BioBERT), clinical generalized autoregressive pretraining for language understanding (Clinical XLNet), label tree-based attention-aware deep model for high-performance extreme multilabel text classification (AttentionXLM), and word-to-vector (Word2Vec) to predict ICD-10-CM. To compare different preprocessing methods for ICD-10-CM, we included DD, medical history, and comorbidity and complication as inputs. We compared the performance of ICD-10-CM prediction using different preprocesses, including definition training, external cause code removal, number conversion, and combination code filtering. For the ICD-10 PCS, the model was trained using different combinations of DD, surgical method, and key words of special examination. The micro F_1_ score and the micro area under the receiver operating characteristic curve were used to compare the model’s performance with that of different preprocessing methods.

**Results:**

BioBERT had an F_1_ score of 0.701 and outperformed other models such as Clinical XLNet, AttentionXLM, and Word2Vec. For the ICD-10-CM, the model had an F_1_ score that significantly increased from 0.749 (95% CI 0.744-0.753) to 0.769 (95% CI 0.764-0.773) with the ICD-10 definition training, external cause code removal, number conversion, and combination code filter. For the ICD-10-PCS, the model had an F_1_ score that significantly increased from 0.670 (95% CI 0.663-0.678) to 0.726 (95% CI 0.719-0.732) with a combination of discharge diagnoses, surgical methods, and key words of special examination. With our preprocessing methods, the model had the highest area under the receiver operating characteristic curve of 0.853 (95% CI 0.849-0.855) and 0.831 (95% CI 0.827-0.834) for ICD-10-CM and ICD-10-PCS, respectively.

**Conclusions:**

The performance of our model with the pretrained contextualized language model and rule-based preprocessing method is better than that of the state-of-the-art model for ICD-10-CM or ICD-10-PCS. This study highlights the importance of rule-based preprocessing methods based on coder coding rules.

## Introduction

### Background

The International Classification of Diseases (ICD) aims to systematically record, analyze, interpret, and compare mortality and morbidity data collected in different areas. ICD transforms the diagnosis of diseases and other health problems from text to alphanumeric codes, which are mixed with English letters and numbers [1]. ICD has become an internationally accepted diagnostic classification system for epidemiological research and health management.

The World Health Organization (WHO) introduced the tenth revision of the International Classification of Diseases (ICD-10) in the 1990s to accommodate the increasing number of diagnoses and related health problems [1]. The clinical modification (CM) and procedure coding system (PCS) of ICD-10 (ICD-10-CM and ICD-10-PCS) have been developed to describe more clinical details with increasing diagnosis and procedure codes and applied in payment methodologies, such as disease-related groups in the United States [2,3]. The transition from ICD-9 to ICD-10-CM or ICD-10-PCS expanded the number of codes. There are only approximately 14,000 diagnosis codes and 3800 procedure codes in ICD-9, but approximately 69,000 in ICD-10-CM and 72,000 in ICD-10-PCS [3]. The expanded codes suppress productivity and increase the cost of disease coding [4]. In practice, the disease coder spent more time interpreting the text of the medical records to ensure the correctness of the disease [4].

The speed and correctness of the classification of the disease coder will be affected by incomplete medical records, orders of diagnosis, undetailed surgical findings, and fragmented exam reports. In addition, hospitals must increase their accuracy in terms of reimbursement. The research found that income can be increased by approximately 5% with a clinician-auditor review in patients discharged following an emergency admission [5].

### Related Work

In recent years, text classification from electronic health records (EHR) data has been widely studied in natural language processing [6], which is a subdiscipline in the fields of artificial intelligence and linguistics. This field explores how to process and use natural language by computers into meaningful representations and maintain the relationships of meanings according to the purpose [7]. Text classification can be divided into the 3 categories of binary, multiclass, and multilabel. Among these, multilabel text classification outputs multiple labels with one or more classes. The multilabel classification task is more challenging because the number of possible combinations of results is greater if the label set is larger.

Teng et al [8] recently proposed a model predicting ICD-10-CM using a medical topic mining method and a cross-textual attentional neural network. It had an F_1_ score of 0.96 in a single label of “atrial fibrillation.” However, even with the same methods proposed to predict the top 50 most frequent ICD-10-CM codes, their model had an F_1_ score of 0.68. This shows that multilabel classification is more complicated than single-label classification. Multilabel classification for ICD-10-PCS is even more challenging owing to its sparsity. Subotin et al [9] proposed a model with code co-occurrence propensity, which improved the prediction of ICD-10-PCS with an F_1_ score from 0.50 to 0.56.

### Previous Work

To facilitate the laborious and time-consuming work process, we have shown that the ICD-10 autocoding system achieved an F_1_ score of 0.67 and 0.58 in CM and PCS by applying word-to-vector (Word2Vec) [10]. Furthermore, we achieved a better F_1_ score of 0.72 and 0.62 in CM and PCS through bidirectional encoder representations from transformers (BERT). In addition, an attention mechanism was used in this classification model to visualize the importance of words used to train new disease coders [11].

In our previous work, some problems were encountered, such as handling the following issues. Some meaningful numbers used in medical terms were removed from the data sets in the preprocessing stage. The combination codes comprising 2 diagnoses in 1 code were hard to be predicted. Other than discharge diagnoses, information from the discharge records was not efficiently included, such as medical history, comorbidity, and complication. In addition, because the writing of medical records was different from the original ICD-10-CM code definition, training our model with the ICD-10-CM definition may be helpful.

Surgical method records and special examination reports are helpful for disease coders to determine the ICD-10-PCS. However, information from special examination reports is challenging to be extracted because it is mixed with uninformative content, such as ultrasound, radiology, endoscopy, and electroencephalography. Furthermore, information from surgical method records is also essential, but the combination algorithm for these types of documents should be studied.

### Objective

This study focuses on interpreting medical records to tackle the problems mentioned above because we found that the accuracy is limited without a rule-based approach. We propose that we can make our model more accurate by adopting coding rules from experienced disease coders in our preprocess. Therefore, this study aims to establish a contextual language model with rule-based preprocessing methods to develop a more accurate and explainable ICD-10 autocoding system.

## Methods

### Ethical Considerations

This retrospective study was approved by the institutional review board of the Far Eastern Memorial Hospital (109086-F and 110028-F), which waived the requirement for informed consent.

### Data Collection

Data were acquired from the electronic medical records of the Far Eastern Memorial Hospital, a medical center in Taiwan, from January 2018 to December 2020. The collected data included admission date, discharge date, discharge summary, ICD-10-CM codes, and ICD-10-PCS codes. The ground-truth ICD-10-CM or ICD-10-PCS codes were labeled by the disease coders.

### Data Description

We obtained 101,974 documents for ICD-10-CM codes and 105,466 documents for ICD-10-PCS codes. Our discharge summary contains 5 types of documents. The discharge diagnoses (DD) listed the main diagnoses related to this hospitalization. The surgical method (SM) includes a description of the surgical procedures and findings. The special examination (SE) includes ultrasound, radiological, endoscopic, and electroencephalography reports. Medical history (MH) contains the process of developing the present illness and the past medical history. Comorbidity and complications (CC) included complications noted during hospitalization.

**Figure 1:**
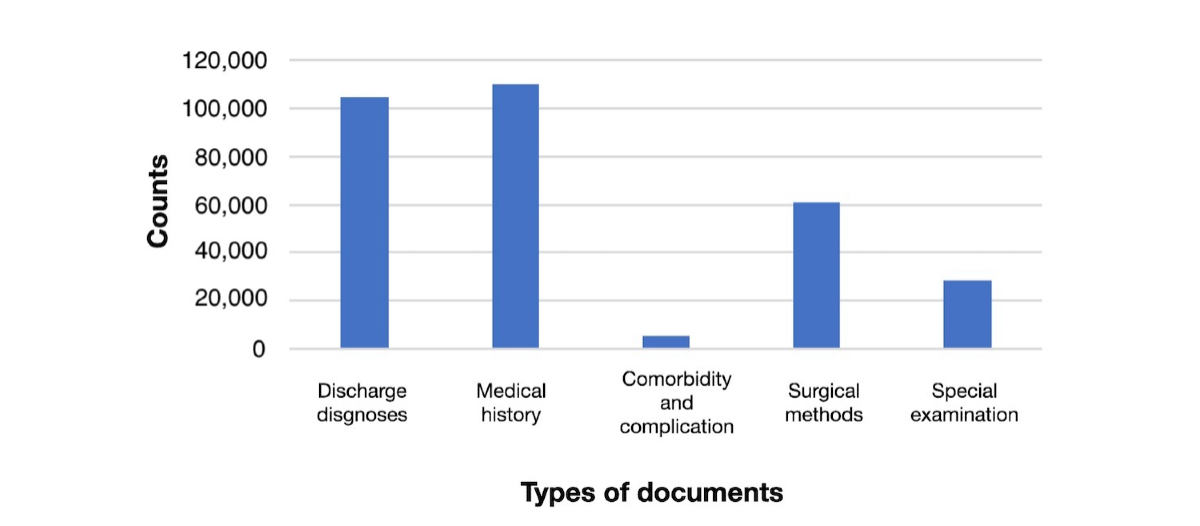
Data counts of 5 types of documents.

Most of these studies included CC and MH (Figure 1). The count of the 3 types of documents in each chapter of the ICD-10-CM and ICD-10-PCS are shown in Multimedia Appendix 1. The chapters were determined by the first 3 codes of the ICD-10 labels annotated by disease coders. The maximal word count was up to 2342 in SE, and the mean word count was up to 149 in MH (Table 1).

**Table 1:** Word counts of 5 types of documents.

Document type	Maximal word count	Mean word count
Discharge diagnoses	480	31
Surgical method	487	11
Special examination	2342	86
Medical history	586	149
Comorbidity and complication	338	5

### Common Text Preprocessing

Null or duplicate data sets and punctuation were removed using the Natural Language Toolkit [12]. Non-English characters were removed before further preprocessing. The text in our EHR was written in mixed English and Chinese. The Chinese part contains the names of the people, places, special customs, and transferred hospital, and is irrelevant to the diagnosis.

### Study Design

We first compared different word embedding methods. Second, we compared the preprocessing methods using the best-performing word embedding methods. To choose the best-performing embeddings, we compared the performance of Word2Vec [13], label tree-based attention-aware deep model for high-performance extreme multilabel text classification (AttentionXLM) [14], biomedical BERT (BioBERT) [15], and clinical generalized autoregressive pretraining for language understanding (clinical XLNet) [16] to predict ICD-10-CM with DD as input. BioBERT had the highest F_1_ score and was chosen to compare the following preprocessing methods for ICD-10-CM or ICD-10-PCS (Multimedia Appendix 2).

**Figure 2:**
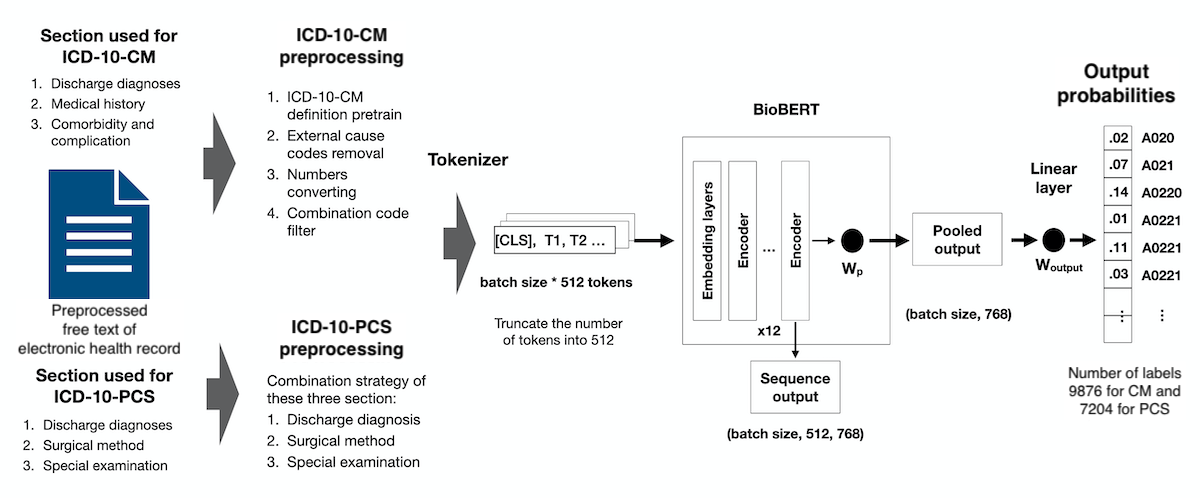
Data processing flow chart and the model architecture. BioBERT: bidirectional encoder representations from transformers for biomedical text mining. CLS: classification; CM: clinical modification; ICD: International Classification of Diseases; PCS: procedure coding system; T: token; Woutput: output weight; Wp: pooled weight.

The sections used for predicting ICD-10-CM were DD, MH, and CC; the sections used for predicting ICD-10-PCS were DD, SM, and SE. The concatenated input text from these sections was long and contained fewer informative components. A proper preprocessing method should be designed to extract helpful information from text. We randomly split the data in a 9:1 ratio into training and validation sets. After the model was trained with the training set, the validation set was used to compare the effects of the following preprocessing methods: the change in the model performance of the trained definition, external cause code removal, number conversion, and combination code filter, which are shown for ICD-10-CM stepwise. The model performance of inputting different document section combinations was compared for ICD-10-PCS, including DD, SM, and SE (Figure 2).

### Model Architecture

After preprocessing, the text was tokenized using the BERT tokenizer. The tokens for BioBERT were truncated to 512 in length because of the model limit [15]. Tokens are then inputted into the BioBERT. A linear layer was connected to the pooled output of BioBERT with labels. The labels are one-hot encodings of all individual ICD-10-CM or ICD-10-PCS codes in our data set, which are 9876 for CM and 7204 for PCS (Figure 2). We calculated the loss by cross entropy. We trained the model using the Adam optimizer and a learning rate of 0.00005 until 100 epochs or met the early stop criteria (less than 0.0001 changes for 10 epochs).

### Data Preprocessing for ICD-10-CM

We included DD, MH, and CC to train the model for ICD-10-CM. We designed a process to include helpful information and remove less informative content. This process contains several components, including the following: MH extraction, CC combining, ICD-10-CM definition training, external cause code removal, number conversion, and combination code filter. The effects of adding the ICD-10-CM definition, external cause code removal, number conversion, and combination code filter on the model performance were compared with the performance before adding these processes.

#### Medical History

We included the MH to extract chronic diseases not mentioned in the DD because we found that some chronic diseases, such as hypertension or chronic kidney disease, were not recorded in approximately 15% of DD in our data. Because the mean length of MH is 5 times that of DD (Table 1), we only extracted key words from MH instead of directly merging DD and MH. We listed these key words and their ICD-10-CM codes in Multimedia Appendix 3. These key words were produced after discussions with disease coders. Only key words found in the text in the MH will be retained for combination after the key word extractor is used.

#### Comorbidity and Complication Combining

Although CC is null in smoothly discharged patients, it affects the ICD-10-CM code if it is not null. ICD-10-CM codes that are frequently inferred from CC include nausea, vomiting, diarrhea, fatigue, and pneumonia. The mean length of the CC was only one-sixth of the DD (Table 1), and thus we combined DD with CC directly.

#### ICD-10-CM Definition Trained

We initiated our model with weights from BioBERT and trained the model on the official ICD-10-CM definition by the WHO as the input and the respective ICD-10-CM code as the output [1]. The model was trained for 100 epochs with early stop criteria (less than 0.0001 changes for 10 epochs). For example, if the output ICD-10-CM code is N39.0, the input text is “urinary tract infection, site not specified.”.

#### External Cause Codes Removal

External cause codes (V01-Y98) define environmental events, circumstances, and conditions, such as the cause of injury, poisoning, and other adverse effects related to an injury. However, it is challenging for a model to predict external cause codes because relevant information is seldom recorded. Because external cause codes do not affect the final disease-related group payment, we removed them from our labels.

#### Number Converting

There are numbers in our EHR, such as the date of the MH, the report’s physiological value, and the header of each line. They were removed because most of them were not informative for our classification task. However, we found that some numbers may affect the ICD-10-CM or ICD-10-PCS prediction, such as pregnancy weeks (“36 weeks gestation of pregnancy”), stage of chronic diseases (“stage 4 chronic kidney disease”), type of disease (“type 2 diabetes mellitus”), and grade of disease (“follicular lymphoma grade 1” and “modified Rankin scale 0”). Thus, we converted all the known essential numbers back to alphabets, such as “stage four chronic kidney disease,” “type two diabetes mellitus,” and “thirty-six weeks gestation of pregnancy,” before removing all numbers.

#### Combination Code Filter

A combination code represents the diagnosis of one or more comorbidities. For example, hypertension with various comorbidities refers to different combinations of codes. To solve these problems, we designed a combination code filter (Multimedia Appendix 4). If the input text contains “hypertension,” it will check whether this case has chronic kidney disease and heart failure. If yes, the combination code filter replaces the original text with the definition of the combination code. In this manner, we prevented the model from providing 2 codes instead of using combination codes.

#### Illustrating Preprocessing for Models Predicting ICD-10-CM

**Figure 3:**
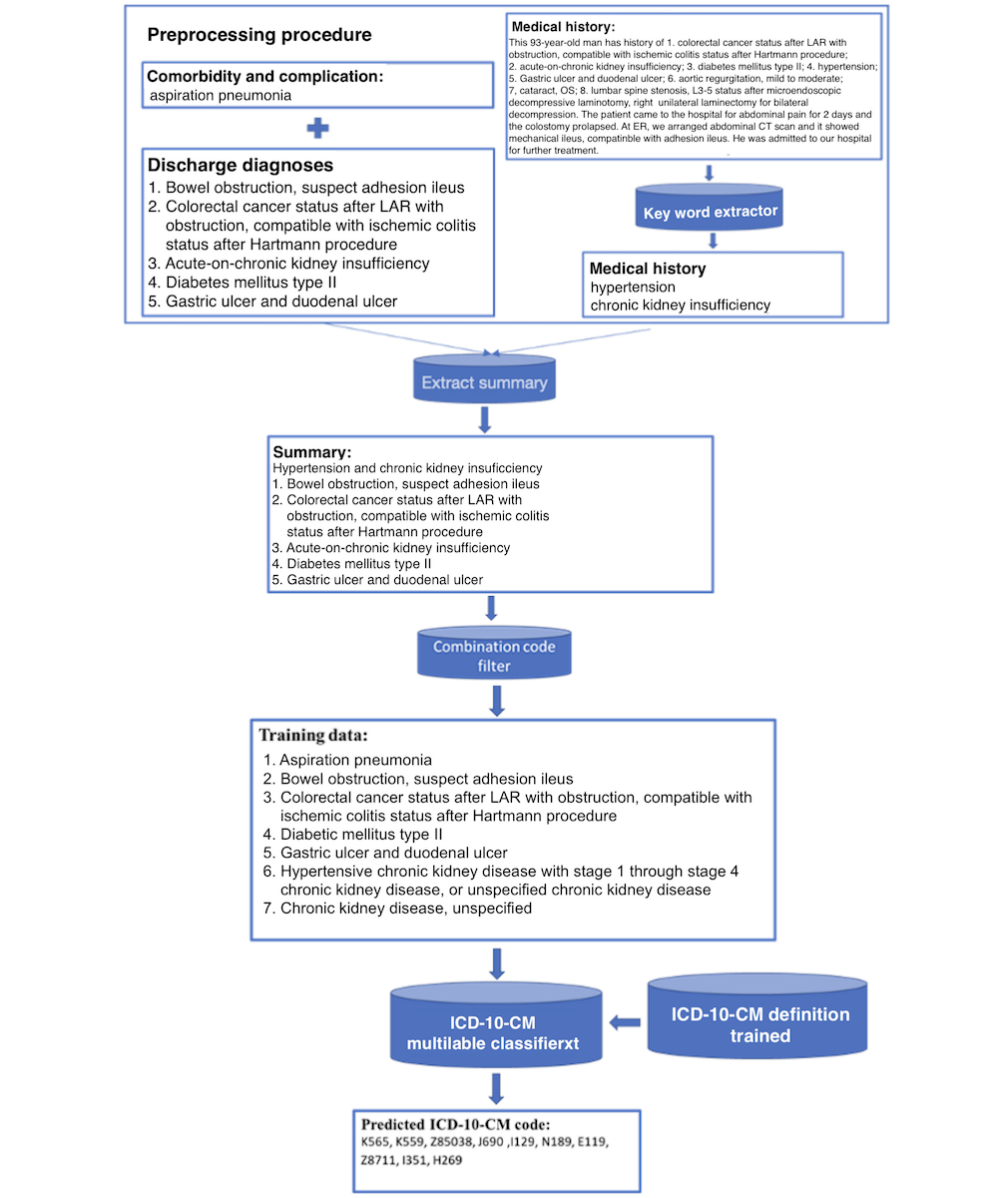
Data preprocessing framework of ICD-10-CM classification model. CM: clinical modification; CT: computed tomography; ER: emergency room; ICD: International Classification of Diseases; L: lumbar; LAR: low anterior resection; OS: oculus sinister.

An example of preprocessing the input data for the models predicting ICD-10-CM is shown in Figure 3. After number conversion, we combined DD with extracted key words from MH, such as “hypertension” and “chronic kidney insufficiency,” into the extract summary. We then transformed the summary using a combination code filter into the training data. We first trained our model using the ICD-10-CM definition and then trained it on the training data.

### Data Preprocessing for ICD-10-PCS

We included DD, SM, and SE to train the model for the ICD-10-PCS. In addition to DD, SM and SE provide helpful information for determining ICD-10-PCS. We trained the model with DD alone, SM alone, and 3 strategies for combining DD with SM and SE, and then compared their performances.

#### Surgical Method

The mean length of SM was one-third of that of DD (Table 1). SM was recorded only if the patient underwent major procedures. To extract the most helpful information for training our model, we proposed a combination of DD and SM.

#### Special Examination

The mean length of SE was 3 times that of DD (Table 1). In an SE report, not all examinations will have the corresponding ICD-10-PCS codes, such as radiological examination or electroencephalography. Therefore, these components should be removed accordingly.

We designed a key word extractor to extract helpful information from SE and to avoid excessive text length. We listed these key words and their ICD-10-PCS codes from high to low frequency in Multimedia Appendix 5. These key words were produced by a discussion with the disease coders. Only key words found in the text in the SE were retained after the key word extractor was used.

After extracting the key words from the SE, we used 2 different combination strategies. First, we input the DD only if the patient has no SM or SE. In the second method, we input the DD if the patient had no SM and added key words from the SE.

#### Illustrating Preprocessing for Models Predicting ICD-10-PCS

**Figure 4:**
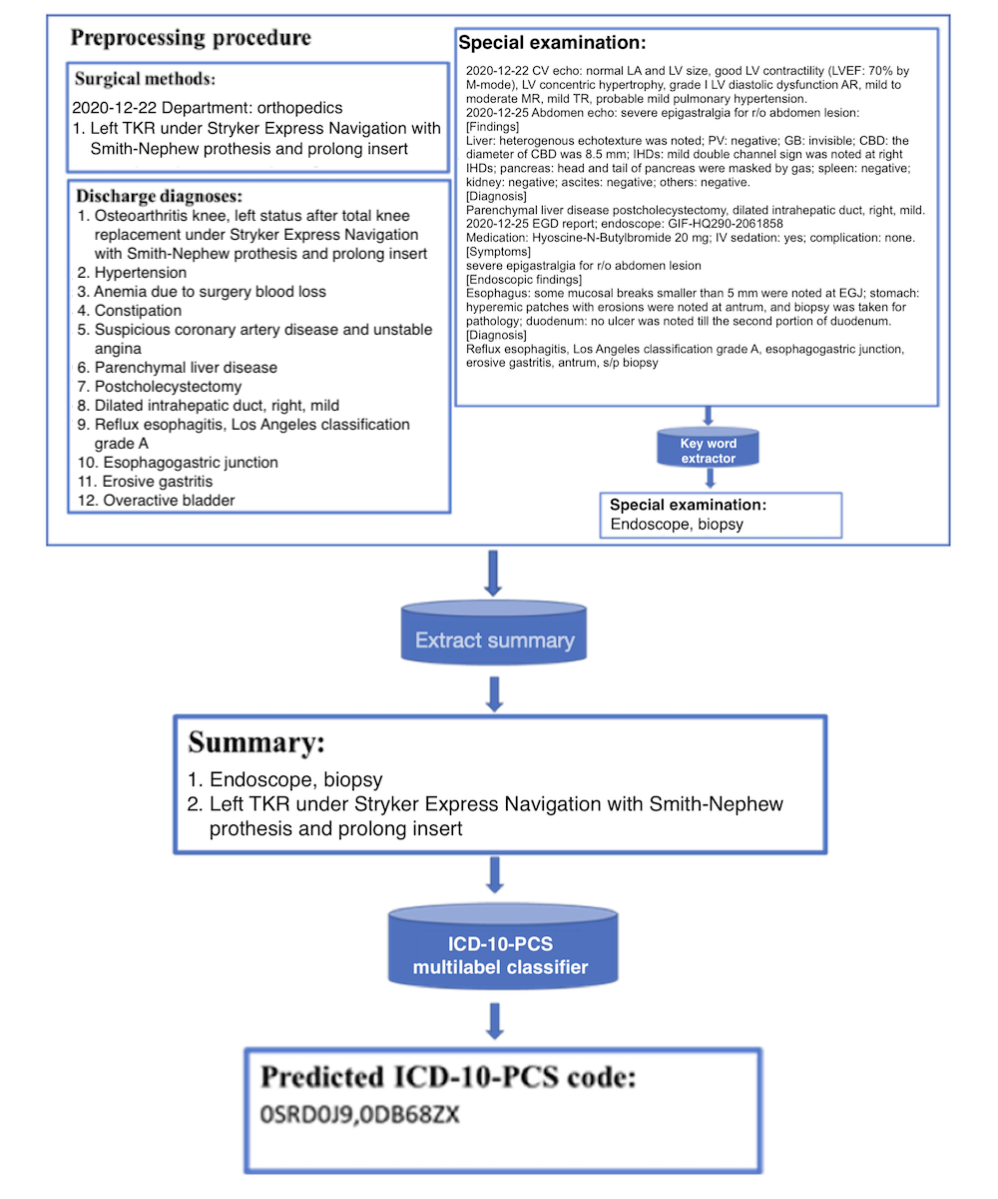
Data preprocessing framework of ICD-10-PCS classification model. AR: aortic regurgitation; CAD: coronary arterial disease; CBD: common bile duct; CV: cardiovascular; EGD: esophagogastroduodenoscopy; EGJ: esophago-gastric junction; GB: gall bladder; ICD: International Classification of Diseases; IHD: intrahepatic duct; IV, intravenous; LA: left atrium; LV: left ventricle; LVEF: left ventricular ejection fraction; MR: mitral regurgitation; PCS: procedure coding system; PV: portal vein; R/O: rule out; s/p: status post; TKR: total knee replacement; TR: tricuspid regurgitation.

An example of preprocessing the input data for models predicting ICD-10-PCS is shown in Figure 4. We first combined DD with extracted key words from SE, such as “endoscope” and “biopsy,” into the extract summary. We then trained our model on these data to predict ICD-10-PCS.

### Preprocessing for ICD-10-CM Label Classification

To compare different preprocessing methods for ICD-10-CM, we included DD, MH, and CC as inputs. We compared the performance of ICD-10-CM prediction using different preprocesses, including definition training, external cause code removal, number conversion, and combination code filtering.

### Preprocessing for ICD-10-PCS Label Classification

In the ICD-10-PCS part of this study, DD, SM, and SE were included as inputs. We compared the prediction performance of the input text, including only DD, SM, and the 3 combination strategies. Combination strategy 1, “SM or DD”—we input the DD only if the case has no SM. Combination strategy 2, “(SM+SE) or DD”—we input the DD only if the case has no SM or SE. Combination strategy 3, “(SM+SE) or (CD+SE)”—we only input DD if the case has no SM and add key words of SE.

### Evaluation Metrics

Microprecision is the summation of true positives divided by the summation of all predicted positive cases (Formula 1). Microrecall is the summation of true positives divided by the summation of all actual positive cases (Formula 2). The micro F_1_ score is the harmonic mean of the microrecall and microprecision, and it is an overall measure of the quality of a classifier’s predictions (Formula 3). The area under the receiver operating characteristic curve (AUROC) was calculated by taking the true-positive rate against the false-positive rate. The micro-average calculates the metrics globally by considering each element of the label indicator matrix as a label. We chose the micro F_1_ score and micro-AUROC to compare the model performance. The F_1_ score, precision, recall, and AUROC are bootstrapped 100 times to calculate the 95% confidence interval.



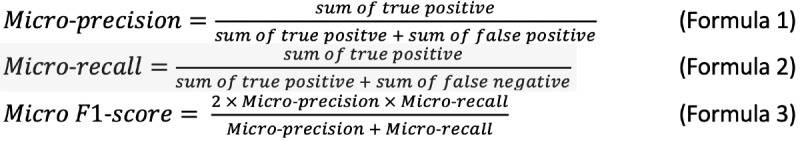



## Results

### ICD-10-CM Label Classification

In our ICD-10-CM multilabel text classification task, each case contained approximately 1 to 20 codes from A00 to Z99. The label set was 9876 in the CM. In the comparison of different embedding models, BioBERT, Clinical XLNet, AttentionXLM, and Word2Vec had the F_1_ score of 0.701, 0.685, 0.654, and 0.651, respectively. The BioBERT model had the highest F_1_ score and was selected for the following experiment. Table 2 shows a comparison of the different preprocessing methods for the ICD-10-CM. The baseline model had a micro F_1_ score of 0.749 (95% CI 0.744-0.753). After the model was trained with the definition, it had an F_1_ score of 0.759 (95% CI 0.754-0.763). After removing the external cause codes, converting the number to the alphabet, and applying a combination code filter, the model had an F_1_ score of 0.763 (95% CI 0.759-0.767), 0.767 (95% CI 0.761-0.772), and 0.769 (95% CI 0.764-0.773), respectively. The baseline model had the AUROC of 0.839 (95% CI 0.835-0.842). With all the preprocessing methods used, the model had an AUROC of 0.858 (95% CI 0.849-0.855).

**Table 2:** Comparison of different preprocessing methods for BioBERT^a^ model on ICD^b^-10-CM^c^. Preprocessing methods are added one by one and 95% CIs are calculated by bootstrapping.

Preprocessing method	Micro F_1_ score (95% CI)	Microprecision (95% CI)	Microrecall (95% CI)	AUROC^d^ (95% CI)
Baseline	0.749 (0.744-0.753)	0.836 (0.832-0.840)	0.678 (0.672-0.684)	0.839 (0.835-0.842)
+Trained with definition	0.759 (0.754-0.763)	0.833 (0.829-0.838)	0.696 (0.690-0.702)	0.848 (0.845-0.851)
+External cause codes removal	0.763 (0.759-0.767)	0.843 (0.840-0.846)	0.697 (0.691-0.702)	0.849 (0.846-0.851)
+Number converting	0.767 (0.761-0.772)	0.845 (0.840-0.849)	0.702 (0.695-0.708)	0.851 (0.847-0.854)
+Combination code filter	0.769 (0.764-0.773)	0.845 (0.841-0.850)	0.706 (0.699-0.711)	0.853 (0.849-0.855)

^a^BioBERT: bidirectional encoder representations from transformers for biomedical text mining.

^b^ICD: International Classification of Diseases.

^c^CM: clinical modification.

^d^AUROC: area under the receiver operating characteristic curve.

### ICD-10-PCS Label Classification

In our ICD-10-PCS multilabel text classification task, each case contained approximately 1-20 codes. The label set was 7204 in the PCS. Table 3 shows a comparison of different input document combinations for the ICD-10-PCS. The models trained with only DD and SM had an F_1_ score of 0.670 (95% CI 0.663-0.678) and 0.618 (95% CI 0.607-0.627), respectively. The model trained with combination strategies 1 (SM or DD), 2 ([SM+SE] or DD), and 3 ([SM+SE] or [DD+SE]) had an F_1_ score of 0.714 (95% CI 0.708-0.721), 0.724 (95% CI 0.718-0.730), and 0.726 (95% CI 0.719-0.732), respectively. The models trained with only DD had the AUROC of 0.800 (95% CI 0.796-0.805). With combination strategy 3, the model had the highest AUROC of 0.831 (95% CI 0.827-0.834).

**Table 3:** Comparison of different preprocessing methods for BioBERT^a^ model on ICD^b^-10-PCS^c^. The 95% CIs are calculated by bootstrapping.

Preprocessing method	Micro F_1_ score (95% CI)	Microprecision (95% CI)	Microrecall (95% CI)	AUROC^d^ (95% CI)
DD^e^	0.670 (0.663-0.678)	0.756 (0.750-0.761)	0.601 (0.593-0.610)	0.800 (0.796-0.805)
SM^f^	0.618 (0.607-0.627)	0.750 (0.741-0.762)	0.524 (0.512-0.534)	0.762 (0.756-0.767)
SM or DD	0.714 (0.708-0.721)	0.790 (0.784-0.791)	0.651 (0.644-0.660)	0.826 (0.822-0.830)
(SM+SE^g^) or DD	0.724 (0.718-0.730)	0.801 (0.794-0.808)	0.661 (0.654-0.668)	0.830 (0.827-0.834)
(SM+SE) or (DD+SE)	0.726 (0.719-0.732)	0.803 (0.797-0.810)	0.661 (0.654-0.669)	0.831 (0.827-0.834)

^a^BioBERT: bidirectional encoder representations from transformers for biomedical text mining.

^b^ICD: International Classification of Diseases.

^c^PCS: procedure coding system.

^d^AUROC: area under the receiver operating characteristic curve.

^e^DD: discharge diagnoses.

^f^SM: surgical method.

^g^SE: special examination.

## Discussion

### Principal Findings

In our study of the multilabel text classification of ICD-10-CM or ICD-10-PCS, each case contained 1-20 codes, and the label set contained up to 9876 and 7204 in CM and PCS, respectively. In our previous study, the model had an F_1_ score of 0.71 and 0.62 in ICD-10-CM and ICD-10-PCS [11]. In this study, we proposed preprocessing methods for ICD-10-CM and ICD-10-PCS, respectively. For the ICD-10-CM, the model had a significant F_1_ score increase from 0.749 (95% CI 0.744-0.753) to 0.769 (95% CI 0.764-0.773) and a significant AUROC increase from 0.839 (95% CI 0.835-0.842) to 0.853 (95% CI 0.849-0.855). For the ICD-10-PCS, the model had an F_1_ score that significantly increased from 0.670 (95% CI 0.663-0.678) to 0.726 (95% CI 0.719-0.732) and an AUROC that significantly increased from 0.800 (95% CI 0.796-0.805) to 0.831 (95% CI 0.827-0.834).

In our comparison of different word embedding methods for ICD-10-CM classification, BioBERT achieved the highest F_1_ score of 0.701 among all embedding methods. This result is consistent with previous research that contextualized representations (BERT and XLNet) showing consistent improvement over noncontextualized models (Word2Vec and AttentionXLM) in multilabel text classification tasks [17]. BioBERT was pretrained on PubMed abstracts and PubMed Central full-text articles to improve the performance of biomedical text-mining tasks [15]. Previous studies confirmed that BioBERT outperformed other embedding methods in classifying ICD-10-CM [11,18].

Training the model with the ICD-10-CM definition increased its F_1_ score from 0.749 to 0.759 (1.3%). Each ICD-10-CM code has a textual description of the definition on the WHO website [1]. Although the text in medical records is different from the WHO’s definition, its semantics should approximate that definition. The results showed that training with definition increased the model performance for the multilabel classification of clinical text. External cause code removal increases the model’s F_1_ score from 0.759 to 0.763 (0.5%). The improvement is limited because external cause codes only accounted for 2.73% (2787/101,974) of our cases.

The number conversion increased the model’s F_1_ score from 0.763 to 0.767 (0.5%). Number converting affected 33.3% (33,978/101,974) of our cases. Retaining informative numbers such as disease type, grade, stages, and pregnancy weeks helps the model learn the relation of these numbers to the different codes. For example, there were differences between type 1 diabetes mellitus (E10) and type 2 diabetes mellitus (E11), follicular lymphoma grades I (C82.0) and II (C82.1), chronic kidney disease stages 1 (N18.1) and 4 (N18.4), and full-term uncomplicated delivery (O80) and preterm delivery (060). The combination code filter increases the model’s F_1_ score from 0.767 to 0.769 (0.2%). The rules of the combination code are challenging to learn through machine learning because this text may be linked to 2 different codes instead of 1 combination code. With all preprocessing methods, the F_1_ score increased from 0.749 to 0.769 (2.6%). Our result is better than the state-of-the-art model of ICD-10-CM with an F_1_ score of 0.68 [8] because we designed a key word extractor and trained our model with ICD-10-CM definition, external cause code removal, number conversion, and combination code filter.

The trained model had the F_1_ score of 0.670 and 0.618 for DD and SM, respectively. DD is more informative for predicting ICD-10-PCS than SM when used alone. However, the model trained using combination strategy 1 (SM or DD) had an F_1_ score of 0.714. The F_1_ score was 6.6% and 15.5% higher than that of DD alone and SM alone, respectively. The F_1_ score of the model trained with SM alone was lower than that of the model trained with DD alone because only 58% (60,558/104,411) of the cases had SM compared to cases with DD. If a patient underwent surgery, the ICD-10-PCS codes were coded according to the SM records. The model trained with combination strategies 2 ([SM+SE] or DD) and 3 ([SM+SE] or [DD+SE]) had an F_1_ score of 0.724 and 0.726, respectively. Their F_1_ scores were 1.4% and 1.7% higher than those of Strategy 1. Adding SE to SM or DD is effective in improving the model performance because several ICD-10-PCS codes are coded according to ultrasound or endoscopic reports in SM. Our result is better than the state-of-the-art model of ICD-10-PCS with an F_1_ score of 0.56 [9] because we designed a key word extractor and combined DD with SM and SE.

### Limitations

Our study had some limitations. First, the data were obtained from a single medical center. Writing habits and disease prevalence may vary between hospitals. Different purposes of coding in different areas may also affect the labels. External validation should be conducted in future studies. Second, although we attempted to include most of the content from the health record, other parts may also contribute to the prediction, such as problem lists and progress notes. Further studies are required to manage these issues.

### Conclusions

ICD-10-CM and ICD-10-PCS codes are widely applied in surveillance, clinical research, and reimbursement. Because of the complexity of ICD-10-CM and ICD-10-PCS, it takes approximately 40.4 min for a record to be coded into ICD-10-CM or ICD-10-PCS manually [2]. This study proposed a model with a combination of a pretrained contextualized language model and rule-based preprocessing methods that outperformed the state-of-the-art models in predicting ICD-10-CM or ICD-10-PCS. This study highlights the importance of rule-based preprocessing methods based on coder coding rules. In EHR, other documents are read manually to determine ICD-10-CM or ICD-10-PCS codes, such as radiology reports, laboratory data, and the problem list. An effective preprocessing method to include documents can be studied in the future.

## Appendix

Multimedia Appendix 1 Counts of types of documents in each chapter of ICD-10-CM and procedure coding system (PCS). CM: clinical modification; ICD: International Classification of Diseases.

Multimedia Appendix 2 Comparing performance and hyperparameters of different embedding models.

Multimedia Appendix 3 ICD-10-CM codes with key words in medical history. CM: clinical modification; ICD: International Classification of Diseases.

Multimedia Appendix 4 Hypertension-related combination code and amount.

Multimedia Appendix 5 ICD-10-PCS codes with key words in special examination. ICD: International Classification of Diseases; PCS: procedure coding system.

## Abbreviations

AttentionXLM: label tree-based attention-aware deep model for high-performance extreme multi-label text classification
AUROC: area under the receiver operating characteristic curve
BERT: bidirectional encoder representations from transformers
BioBERT: bidirectional encoder representations from transformers for biomedical text mining
CC: comorbidity and complications
CM: clinical modification
DD: discharge diagnoses
EHR: electronic health records
ICD: International Classification of Diseases
MH: medical history
PCS: procedure coding system
SE: special examination
SM: surgical method
WHO: World Health Organization
Word2Vec: word to vector
XLNet: generalized autoregressive pretraining for language
