# Supplementary material for: Automatic International Classification of Diseases Coding System: Deep Contextualized Language Model With Rule-Based Approaches
Source: JMIR Med Inform. 2022 Jun 29;10(6):e37557. doi: 10.2196/37557 (PMC9282222; doi:10.2196/37557)

**Table S1.** Counts of three types of documents in each chapter of ICD-10-CM

| Chapter | Discharge diagnoses | Medical history | Comorbidity and complication |
| --- | --- | --- | --- |
| A00-B99 | 21,947 | 24,483 | 1,677 |
| C00-D49 | 34,330 | 38,127 | 3,673 |
| D50-D89 | 10,368 | 11,774 | 1,028 |
| E00-E90 | 50,184 | 58,159 | 2,886 |
| F00-F99 | 8,711 | 9,444 | 336 |
| G00-G99 | 8,874 | 9,996 | 632 |
| H00-H59 | 2,404 | 2,625 | 116 |
| H60-H95 | 1,601 | 1,795 | 84 |
| I00-I99 | 79,426 | 92,760 | 5,595 |
| J00-J99 | 47,547 | 54,369 | 2,769 |
| K00-K93 | 44,508 | 50,651 | 2,772 |
| L00-L99 | 7,244 | 8,137 | 489 |
| M00-M99 | 12,575 | 14,032 | 743 |
| N00-N99 | 38,921 | 43,687 | 2,240 |
| O00-O99 | 5,605 | 8,203 | 392 |
| P00-P96 | 3,755 | 4,170 | 641 |
| Q00-Q99 | 1,722 | 2,151 | 174 |
| R00-R99 | 21,274 | 24,154 | 2,237 |
| S00-T98 | 27,019 | 31,166 | 2,253 |
| V01-Y98 | 12,577 | 14,294 | 822 |
| Z00-Z99 | 41,108 | 49,572 | 2,728 |
| U00-U99 | 150 | 219 | 0 |
| Pseudo code | 264 | 2,803 | 48 |

**Figure S1.** Data distribution in each chapter of ICD-10-CM.


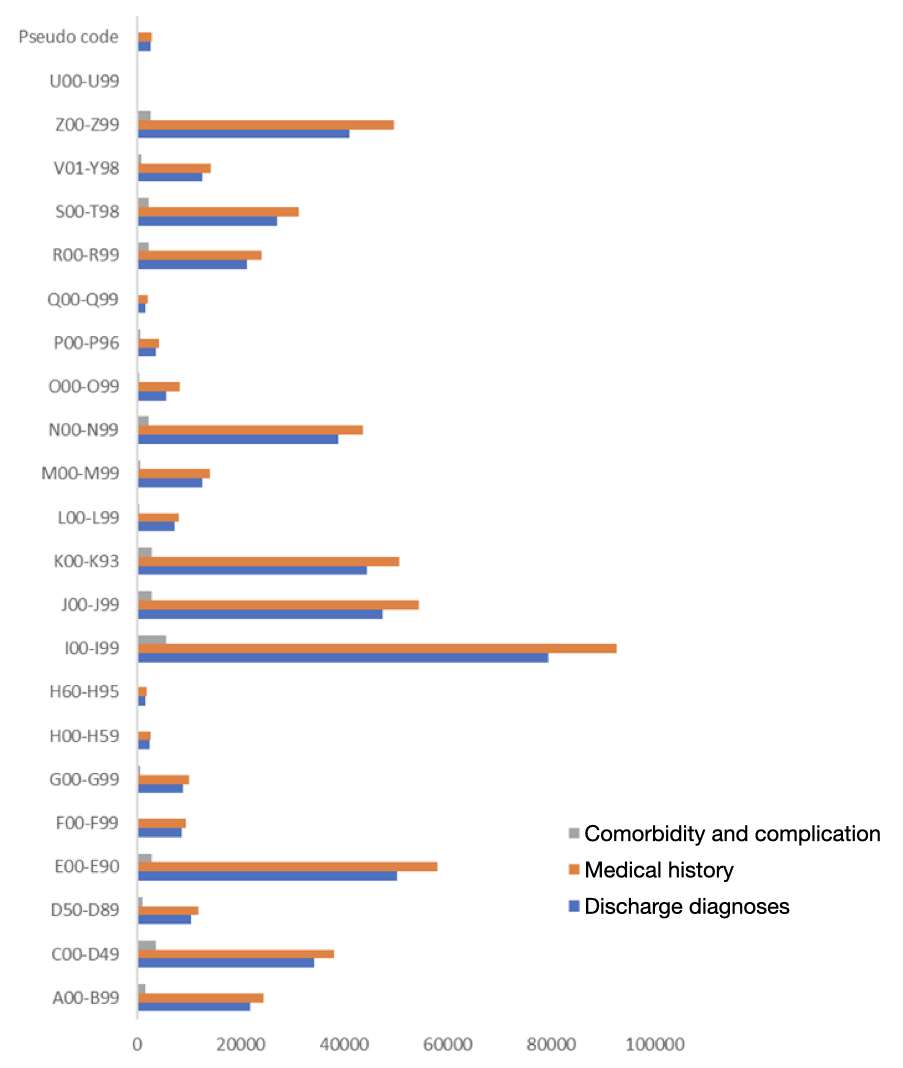


**Table S2.** Counts of three types of documents in each chapter of ICD-10-PCS

| Chapter | Discharge diagnoses | Special examination | Surgical method |
| --- | --- | --- | --- |
| 0 | 187,178 | 42,029 | 133,676 |
| 1 | 8,200 | 29 | 2,655 |
| 2 | 365 | 245 | 306 |
| 3 | 19,075 | 2,344 | 2,570 |
| 4 | 13,203 | 9,461 | 3,538 |
| 5 | 24,867 | 12,490 | 11,946 |
| 6 | 1,263 | 527 | 70 |
| 7 | 0 | 0 | 0 |
| 8 | 411 | 14 | 315 |
| 9 | 0 | 0 | 0 |
| B | 24,774 | 16,129 | 6,840 |
| C | 251 | 4 | 3 |
| D | 1,904 | 175 | 439 |
| E | 0 | 0 | 0 |
| F | 269 | 123 | 14 |
| G | 0 | 0 | 0 |

**Figure S2.** Data distribution in each chapter of ICD-10-PCS.


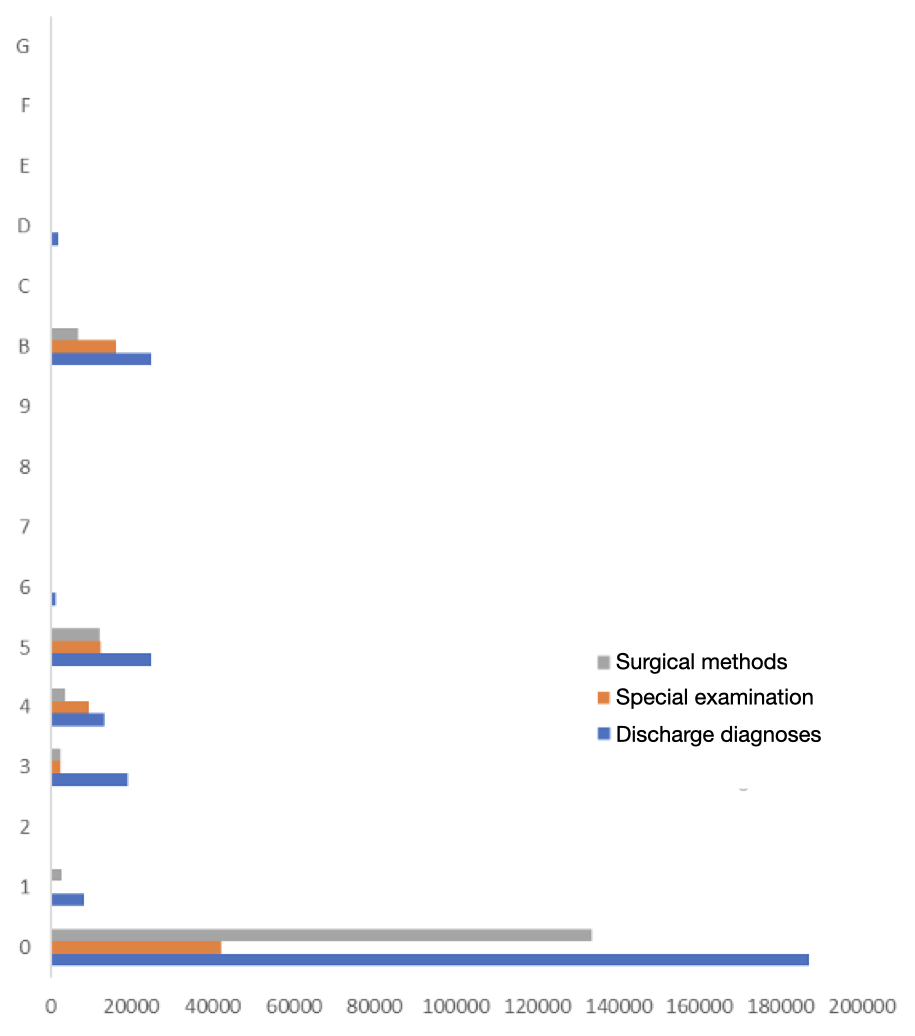

Supplement: Multimedia Appendix 1 [file medinform_v10i6e37557_app1.docx]
