# Supplementary material for: Automatic International Classification of Diseases Coding System: Deep Contextualized Language Model With Rule-Based Approaches
Source: JMIR Med Inform. 2022 Jun 29;10(6):e37557. doi: 10.2196/37557 (PMC9282222; doi:10.2196/37557)
Supplement: Multimedia Appendix 2 [file medinform_v10i6e37557_app2.docx]

**Figure S3.** Comparing performance of different embedding models


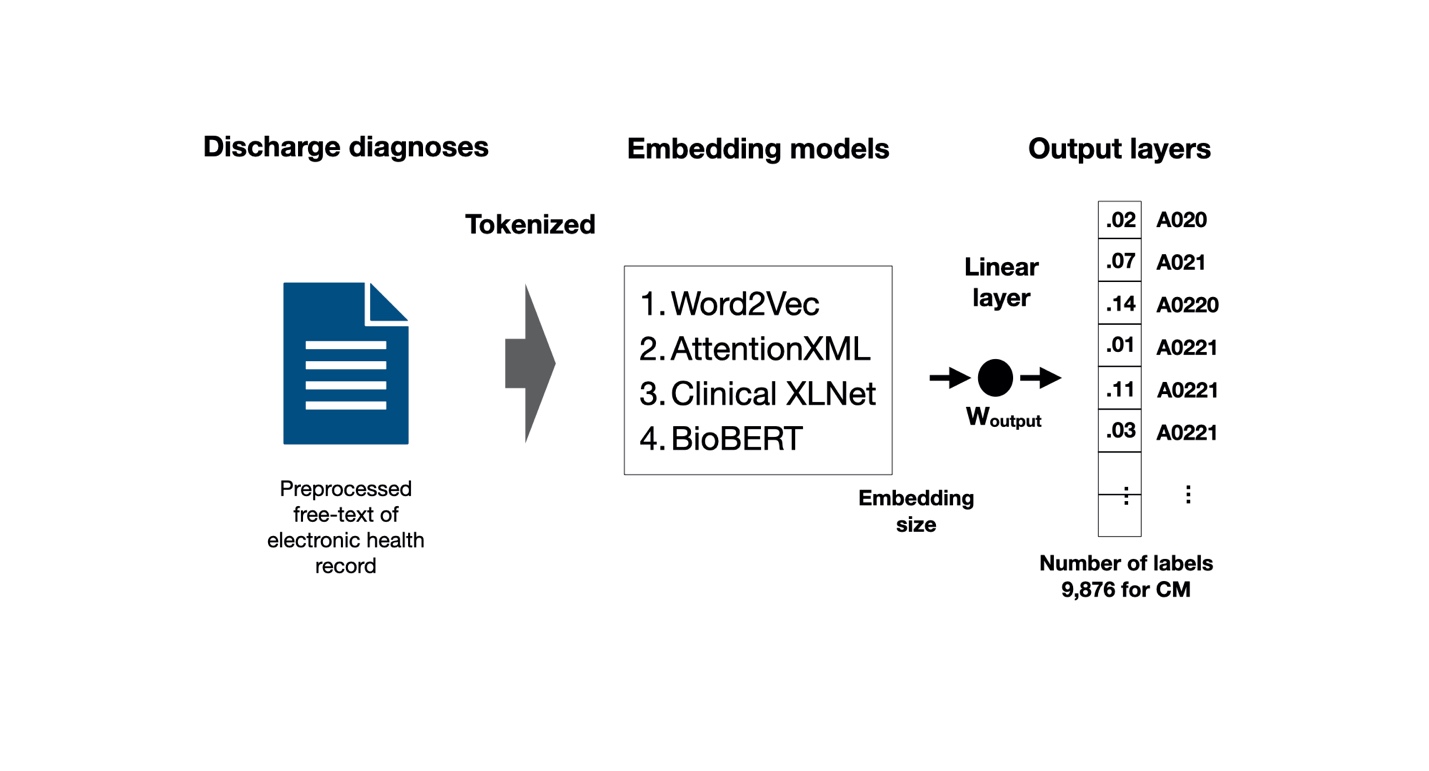


**Table S3.** Hyperparameters of word embedding models

| Model | Hyperparameters | Size/Number |
| --- | --- | --- |
| Word2Vec | word embedding size | 300 |
| AttentionXML | word embedding size | 256 |
| Clinical XLNet | word embedding size | 1,024 |
|  | sentence embedding size | 1,024 |
|  | position embedding size | 1,024 |
|  | intermediate size | 4,096 |
|  | attention head number | 16 |
|  | hidden layer number | 124 |
|  | dropout | 0.1 |
| BioBERT | word embedding size | 768 |
|  | sentence embedding size | 768 |
|  | position embedding size | 768 |
|  | intermediate size | 3,072 |
|  | attention head number | 12 |
|  | hidden layer number | 12 |
|  | dropout | 0.1 |
