# Supplementary material for: Automatic International Classification of Diseases Coding System: Deep Contextualized Language Model With Rule-Based Approaches
Source: JMIR Med Inform. 2022 Jun 29;10(6):e37557. doi: 10.2196/37557 (PMC9282222; doi:10.2196/37557)
Supplement: Multimedia Appendix 3 [file medinform_v10i6e37557_app3.docx]

**Table S4.** ICD-10-CM codes with keywords in medical history. DM, diabetes mellitus; T2DM, type 2 diabetes mellitus; CAD, coronary artery disease; UTI, uninary tract infection; BPH, benign prostatic hyperplasia; CKD, chronic kidney disease; CRF, chronic renal failure; HD, hemodialysis; PD, peritoneal dialysis; Af, atrial fibrillation; GERD, gastro-esophageal reflux disease; BMS, bare metal stent; DES, drug eluting stent; CVA, cerebrovascular accident, TIA, transient ischemic attack; COPD , chronic obstructive pulmonary disease; SCC, squamous cell carcinoma; ICH, intracranial hemorrhage; SCI, spinal cord injury; TB, tuberculosis; ca, cancer.

| **ICD-10-CM code** | **Definition** | **Keyword** | **Number** |
| --- | --- | --- | --- |
| I10 | Essential (primary) hypertension | hypertension; hypertensive | 24,176 |
| E11.9 | Type 2 diabetes mellitus without complications | DM; T2DM; diabetes | 16,357 |
| E78.5 | Hyperlipidemia, unspecified | hyperlipidemia; dyslipidemia | 11,341 |
| I25.10 | Atherosclerotic heart disease of native coronary artery without angina pectoris | CAD; coronay artery disease; atherosclerosis; ischemic heart | 10,319 |
| N39.0 | Urinary tract infection, site not specified | UTI; urinary tract infection; pyuria | 8,826 |
| Z51.11 | Encounter for antineoplastic chemotherapy | chemotherapy | 6,340 |
| N40.0 | Benign prostatic hyperplasia without lower urinary tract symptoms | BPH; prostate | 4,812 |
| N18.9 | Chronic kidney disease, unspecified | CKD; kidney disease; renal disease; CRF | 3,658 |
| N18.6 | End stage renal disease | CKD; uremia; hemodialysis; peritoneal dialysis; HD; PD | 3,631 |
| I48.391 | Unspecified atrial fibrillation | Af; atrial fibrillation | 3,228 |
| K2130 | Gastro-esophageal reflux disease with esophagitis | GERD; heart burn; gastroesophageal reflux | 3,063 |
| I12.9 | Hypertensive chronic kidney disease with stage 1 through stage 4 chronic kidney disease, or unspecified chronic kidney disease | hypertension; hypertensive; kidney disease; CKD; CRF | 2,986 |
| Z95.5 | Presence of coronary angioplasty implant and graft | CAD; stent; BMS; DES | 2,774 |
| Z86.73 | Personal history of transient ischemic attack (TIA), and cerebral infarction without residual deficits | ischemic stroke; old CVA; TIA | 2,483 |
| Z86.79 | Personal history of other diseases of the circulatory system | aneurysm; CAD; heart disease; CVA | 1,542 |
| Z87.11 | Personal history of peptic ulcer disease | gastric ulcer; duodenal ulcer | 1,295 |
| Z87.891 | Personal history of nicotine dependence | chronic obstructive pulmonary disease; COPD; smoker; smoking; cigarette | 714 |
| Z85.3 | Personal history of malignant neoplasm of breast | breast; breast cancer; breast ca | 494 |
| Z87.81 | Personal history of (healed) traumatic fracture | fracture | 479 |
| Z87.442 | Personal history of urinary calculi | ureteral stone; renal stone; stone; ureteral | 454 |
| Z87.19 | Personal history of other diseases of the digestive system | reflux esophagitis; abdomen; ulcer | 420 |
| Z85.818 | Personal history of malignant neoplasm of other sites of lip, oral cavity, and pharynx | wide excision; squamous cell carcinoma; flap reconstruction; flap; buccal SCC | 361 |
| Z85.038 | Personal history of other malignant neoplasm of large intestine | colon cancer; hemicolectomy | 359 |
| Z87.820 | Personal history of traumatic brain injury | head injury; brain injury; traumatic brain hemorrhage; ICH; intracranial hemorrhage | 303 |
| Z87.828 | Personal history of other (healed) physical injury and trauma | burn; SCI; spinal cord injury; eye injury; eye trauma | 280 |
| Z85.41 | Personal history of malignant neoplasm of cervix uteri | cervical cancer; cervical ca | 236 |
| Z86.11 | Personal history of tuberculosis | tuberculosis; old TB; TB | 205 |
| Z86.39 | Personal history of other endocrine, nutritional and metabolic disease | poor appetite; metastasis | 204 |
| Z85.46 | Personal history of malignant neoplasm of prostate | prostate cancer; prostate ca; prostate neoplasm | 159 |
| Z85.048 | Personal history of other malignant neoplasm of rectum, rectosigmoid junction, and anus | rectal cancer; anterior resection; rectal adenocarcinoma; colostomy | 146 |
