# Supplementary material for: Automatic International Classification of Diseases Coding System: Deep Contextualized Language Model With Rule-Based Approaches
Source: JMIR Med Inform. 2022 Jun 29;10(6):e37557. doi: 10.2196/37557 (PMC9282222; doi:10.2196/37557)
Supplement: Multimedia Appendix 4 [file medinform_v10i6e37557_app4.docx]

**Table S5.** Hypertension related combination code and amount

| Combination Code | Definition | Number |
| --- | --- | --- |
| I11.0 | Hypertensive heart disease with heart failure | 1,365 |
| I11.9 | Hypertensive heart disease without heart failure | 956 |
| I12.0 | Hypertensive chronic kidney disease with stage 5 chronic kidney disease or end stage renal disease | 2,266 |
| I12.9 | Hypertensive chronic kidney disease with stage 1 through stage 4 chronic kidney disease, or unspecified chronic kidney disease | 2,666 |
| I13.0 | Hypertensive heart and chronic kidney disease with heart failure and stage 1 through stage 4 chronic kidney disease, or unspecified chronic kidney disease | 751 |
| I13.10 | Hypertensive heart and chronic kidney disease without heart failure, with stage 1 through stage 4 chronic kidney disease, or unspecified chronic kidney disease | 114 |
| I13.11 | Hypertensive heart and chronic kidney disease without heart failure, with stage 5 chronic kidney disease, or end stage renal disease | 99 |
| I13.2 | Hypertensive heart and chronic kidney disease with heart failure and with stage 5 chronic kidney disease, or end stage renal disease | 508 |
|  | sum | 8,725 |
