# Supplementary material for: Automatic International Classification of Diseases Coding System: Deep Contextualized Language Model With Rule-Based Approaches
Source: JMIR Med Inform. 2022 Jun 29;10(6):e37557. doi: 10.2196/37557 (PMC9282222; doi:10.2196/37557)
Supplement: Multimedia Appendix 5 [file medinform_v10i6e37557_app5.docx]

**Table S6.** ICD-10-PCS codes with keywords in special examination. Abbreviations: cath, catheterization; CAD, coronary arterial disease; PES, panendoscopy; 1VD, one vessel disease; DES, drug eluting stent; BMS, bare metal stent; 2VD, two vessel disease; EPS, electrophysical study; RFCA, radiofrequency catheter ablation; TPM, temporary pacemaker; ERBD, endoscopic retrograde biliary drainage; PTGBD, percutaneous transhepatic gallbladder drainage; EVL, endoscopic variceal ligation; IABP, intra-aortic balloon pump.

| **ICD-10-PCS code** | **Definition** | **Keyword** | **Number** |
| --- | --- | --- | --- |
| 4A023N7 | Measurement of Cardiac Sampling and Pressure, Left Heart, Percutaneous Approach | cath; cardiac cath; CAD | 8,346 |
| B2110ZZ | Fluoroscopy of Multiple Coronary Arteries using High Osmolar Contrast | cath; cardiac cath; CAD | 8,019 |
| 0DJ08ZZ | Inspection of Upper Intestinal Tract, Via Natural or Artificial Opening Endoscopic | esophagus endoscopy; PES; panendoscopy; endoscope | 2,427 |
| 027034Z | Dilation of Coronary Artery, One Site with Drug-eluting Intraluminal Device, Percutaneous Approach | 1VD; DES | 2,272 |
| 0W3P8ZZ | Control Bleeding in Gastrointestinal Tract, Via Natural or Artificial Opening Endoscopic | ulcer; bleeding | 991 |
| 02703DZ | Dilation of Coronary Artery, One Site with Intraluminal Device, Percutaneous Approach | 1VD; BMS | 986 |
| 02703ZZ | Dilation of Coronary Artery, One Site, Percutaneous Approach | 1VD | 899 |
| 0DB68ZX | Excision of Stomach, Via Natural or Artificial Opening Endoscopic, Diagnostic | biopsy | 801 |
| 02HV33Z | Insertion of Infusion Device into Superior Vena Cava, Percutaneous Approach | Port-A | 695 |
| 0BJ08ZZ | Inspection of Tracheobronchial Tree, Via Natural or Artificial Opening Endoscopic | bronchoscopy; bronchial | 656 |
| 0W9930Z | Drainage of Right Pleural Cavity with Drainage Device, Percutaneous Approach | right pigtail | 640 |
| 0DJD8ZZ | Inspection of Lower Intestinal Tract, Via Natural or Artificial Opening Endoscopic | colonoscopy | 637 |
| 3E063KZ | Introduction of Other Diagnostic Substance into Central Artery, Percutaneous Approach | electrophysiologic | 502 |
| 0W993ZZ | Drainage of Right Pleural Cavity, Percutaneous Approach | drainage | 498 |
| 027134Z | Dilation of Coronary Artery, Two Sites with Drug-eluting Intraluminal Device, Percutaneous Approach | 2VD; DES | 481 |
| 4A023FZ | Measurement of Cardiac Rhythm, Percutaneous Approach | EPS | 474 |
| 0W9B30Z | Drainage of Left Pleural Cavity with Drainage Device, Percutaneous Approach | left pigtail | 458 |
| 0B110F4 | Bypass Trachea to Cutaneous with Tracheostomy Device, Open Approach | tracheostomy | 421 |
| 02583ZZ | Destruction of Conduction Mechanism, Percutaneous Approach | RFCA | 403 |
| 0W9G3ZZ | Drainage of Peritoneal Cavity, Percutaneous Approach | ascites | 399 |
| 5A1223Z | Performance of Cardiac Pacing, Continuous | TPM | 364 |
| 0F798DZ | Dilation of Common Bile Duct with Intraluminal Device, Via Natural or Artificial Opening Endoscopic | ERBD | 333 |
| 02HK3JZ | Insertion of Pacemaker Lead into Right Ventricle, Percutaneous Approach | pacemaker | 318 |
| 0JH60XZ | Insertion of Vascular Access Device into Chest Subcutaneous Tissue and Fascia, Open Approach | Port-A | 279 |
| 02H63JZ | Insertion of Pacemaker Lead into Right Atrium, Percutaneous Approach | pacemaker | 270 |
| 0JH606Z | Insertion of Pacemaker, Dual Chamber into Chest Subcutaneous Tissue and Fascia, Open Approach | pacemaker | 229 |
| 0F9430Z | Drainage of Gallbladder with Drainage Device, Percutaneous Approach | drainage; PTGBD | 204 |
| 06L34CZ | Occlusion of Esophageal Vein with Extraluminal Device, Percutaneous Endoscopic Approach | EVL | 196 |
| 02713DZ | Dilation of Coronary Artery, Two Sites with Intraluminal Device, Percutaneous Approach | 2VD; BMS | 189 |
| 5A02210 | Assistance with Cardiac Output using Balloon Pump, Continuous | IABP | 174 |
